# Plasma, urine and tissue concentrations of Flunixin and Meloxicam in Pigs

**DOI:** 10.1186/s12917-020-02556-4

**Published:** 2020-09-16

**Authors:** Emma Nixon, Travis P. Mays, Patricia A. Routh, James L. Yeatts, Virginia R. Fajt, Thomas Hairgrove, Ronald E. Baynes

**Affiliations:** 1 Department of Population Health and Pathobiology, College of Veterinary Medicine, North Carolina State University, 27607 Raleigh, NC United States; 2 Texas A & M Veterinary Medical Diagnostic Laboratory, 77840 College Station, TX United States; 3 Department of Veterinary Physiology and Pharmacology, College of Veterinary Medicine and Biomedical Sciences, Texas A & M University, 77843 College Station, TX United States; 4 Texas A & M AgriLife Extension, Texas A & M University, 77843 College Station, TX United States

**Keywords:** Pig, Flunixin, Meloxicam, Pharmacokinetics, NSAIDs, Residue, Urine, Show

## Abstract

**Background:**

The objective of this study was to determine the renal clearance of flunixin and meloxicam in pigs and compare plasma and urine concentrations and tissue residues. Urine clearance is important for livestock show animals where urine is routinely tested for these drugs. Fourteen Yorkshire/Landrace cross pigs were housed in individual metabolism cages to facilitate urine collection. This is a unique feature of this study compared to other reports. Animals received either 2.2 mg/kg flunixin or 0.4 mg/kg meloxicam via intramuscular injection and samples analyzed by mass spectrometry. Pigs were euthanized when drugs were no longer detected in urine and liver and kidneys were collected to quantify residues.

**Results:**

Drug levels in urine reached peak concentrations between 4 and 8 h post-dose for both flunixin and meloxicam. Flunixin urine concentrations were higher than maximum levels in plasma. Urine concentrations for flunixin and meloxicam were last detected above the limit of quantification at 120 h and 48 h, respectively. The renal clearance of flunixin and meloxicam was 4.72 ± 2.98 mL/h/kg and 0.16 ± 0.04 mL/h/kg, respectively. Mean apparent elimination half-life in plasma was 5.00 ± 1.89 h and 3.22 ± 1.52 h for flunixin and meloxicam, respectively. Six of seven pigs had detectable liver concentrations of flunixin (range 0.0001–0.0012 µg/g) following negative urine samples at 96 and 168 h, however all samples at 168 h were below the FDA tolerance level (0.03 µg/g). Meloxicam was detected in a single liver sample (0.0054 µg/g) at 72 h but was below the EU MRL (0.065 µg/g).

**Conclusions:**

These data suggest that pigs given a single intramuscular dose of meloxicam at 0.4 mg/kg or flunixin at 2.2 mg/kg are likely to have detectable levels of the parent drug in urine up to 2 days and 5 days, respectively, after the first dose, but unlikely to have tissue residues above the US FDA tolerance or EU MRL following negative urine testing. This information will assist veterinarians in the therapeutic use of these drugs prior to livestock shows and also inform livestock show authorities involved in testing for these substances.

## Background

Livestock show authorities drug test the urine in show animals in order to ensure fair competition. One of the groups of drugs of importance in this setting is nonsteroidal anti-inflammatory drugs (NSAIDs), which may be used to conceal an injury that would otherwise prevent a show animal from competing. Show animals are also sold soon after a livestock show competition, and there has always been the assumption that if the urine is clear of the drug, then the organs responsible for drug clearance will be free of drug residues. While, urine concentrations may correlate with plasma concentrations, using urine concentrations to assess potential tissue residues could lead to inaccurate predictions of target tissue concentrations and can result in violative drug residues [1–4].

There is only one US FDA-approved NSAID for use in pigs: flunixin meglumine for the control of pyrexia associated with swine respiratory disease. Another commonly prescribed NSAID, meloxicam, is not approved for use in the US in pigs, but is in the EU and Canada. The FDA has an established tolerance for flunixin in pigs, which is 30 part per billion (ppb) in liver (the target tissue) and 25 ppb in muscle [5]. No tolerance has been established for meloxicam in pigs, and therefore detection of any tissue residue would be considered a violation by USDA Food Safety and Inspection Service (FSIS).

There are numerous studies describing flunixin plasma and urine concentrations in cattle, goats, horses, camels and dogs [2, 3, 6–9], as well as pigs [10, 11]. It should also be noted that relationship between plasma and urine has been the background for doping control in horses [8, 12]. Of the two studies that examined flunixin in urine of pigs, only one of those attempted to establish a correlation with tissue concentrations. However, that study used a single spot urine sample taken at necropsy to predict the residue depletion profile in edible tissues. Those results showed that earlier urine samples (24 h) were highly variable in concentration and are affected by many factors, including the urinary output, voiding intervals, last voiding time, postvoid residual urine volume [10]. The author recommended that future studies should consider using metabolism cages to collect cumulative urine samples to improve the prediction of tissue concentrations. This was a primary goal of our current study. The second study [11] calculated 6.8% of the parent drug was excreted in urine following administration of 2.2 mg/kg flunixin intramuscularly once daily for 3 days in 3 month old pigs. However this study did not assess tissue concentrations of flunixin in pigs. There are far fewer data available regarding meloxicam concentration-time profiles in urine in large animal species, with only two studies in horses and goats [2, 13], and no published studies have assessed the relationship between meloxicam concentrations in plasma, tissues and urine in pigs.

The objective of this study was to compare plasma and interstitial fluid; ISF concentrations to urine concentrations of both meloxicam and flunixin, with a focus on estimating the renal clearance of these parent drugs in pigs which has not been reported before. Show animals are often sold for slaughter soon after a livestock show, therefore, another aim of this project was not to substitute tissue residue testing with urine testing, but to be able to inform owners of show animals that even though the urine may be cleared of the drug during the show, there may or may not be violative tissue residues in their show animals.

## Results

All pigs (n = 7 for each treatment) completed the study with no adverse effects. One pig from the pilot study was excluded from the flunixin urine results due to carryover in the collection tray. One pig was excluded from the flunixin plasma and ISF results due to loss of the catheter and ISF probe.

### Flunixin

#### Plasma and ISF

**Fig. 1:**
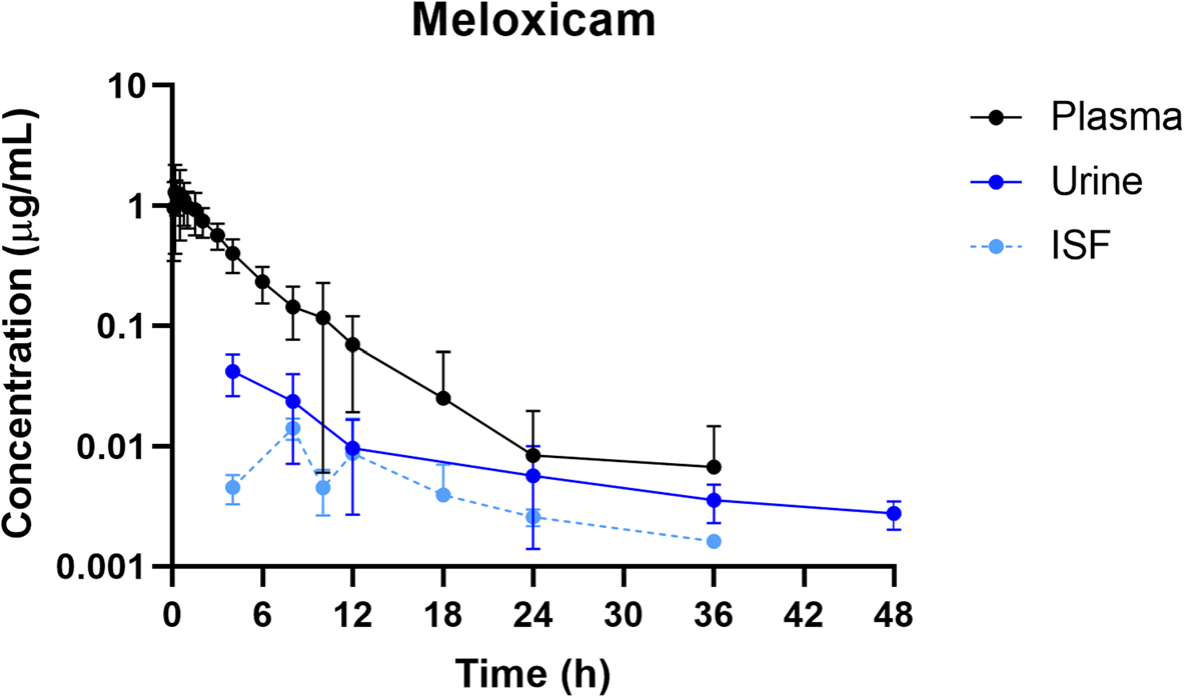
Plasma, urine and interstitial fluid meloxicam concentration-time profiles following intramuscular administration of 0.4 mg/kg meloxicam to pigs. Data are represented as mean ± standard deviation

Mean plasma and ISF flunixin concentrations over time following a single IM injection of 2.2 mg/kg are presented in Fig. 1. Parameters describing the pharmacokinetics of flunixin following a single IM injection are presented in Table 1. Flunixin concentrations in plasma were last detected above the LOQ of 0.0005 µg/mL at 60 h. The plasma pharmacokinetics of flunixin after IM administration were characterized by rapid absorption, an apparent large volume of distribution/F and an apparent elimination half-life which was relatively short. In interstitial fluid, the average free maximum concentration of flunixin (Cmax) was 0.0039 µg/mL at 6 hours (Tmax) after flunixin administration.

**Table 1:** Plasma pharmacokinetic parameters following intramuscular administration of either flunixin meglumine 2.2 mg/kg (n = 6) or meloxicam 0.4 mg/kg (n = 7)

Plasma Pharmacokinetic Parameters					
**Parameter**	**Units**	**Flunixin**		**Meloxicam**	
		**Mean**	**Range**	**Mean**	**Range**
**Dose**	mg/kg	2.20		0.40	
**MRT**	h	5.63	(3.44–8.58)	4.15	(3.23–6.37)
**T**_**1/2**_	h	5.41	(3.01–9.34)	3.34	(2.35–6.37)
**λ**_**z**_	h	0.13	(0.07–0.23)	0.21	(0.11–0.29)
**T**_**max**_	1/h	0.22	(0.08–0.75)	0.37	(0.17–1.50)
**C**_**max**_	µg/mL	2.18	(1.82–2.87)	1.20	(0.75–2.85)
**AUC**_**last**_	h*µg/mL	5.96	(3.95–7.83)	4.63	(3.01–8.55)
**AUC**_**inf**_	h*µg/mL	5.97	(3.97–7.85)	4.65	(3.02–8.67)
**AUC**_**extrap**_	%	0.23	(0.10–0.38)	0.31	(0.10–1.31)
**Vd/F**	L/kg	2.88	(1.22–7.47)	0.41	(0.23–0.58)
**Cl/F**	L/h/kg	0.37	(0.28–0.55)	0.09	(0.05–0.13)

Data are shown as geometric mean and range.* MRT *Mean residence time, T_1/2_; apparent elimination half-life, λz; slope of the terminal phase, *Tmax *Time to maximal concentration, *Cmax *Maximum concentration, *AUC *Area under the concentration-time curve, *Vd/F *Volume of distribution per fraction absorbed, *Cl/F *Total body clearance per fraction absorbed.

#### Urine

The highest concentration of flunixin in urine (Cmax; 1.55 ± 0.96 µg/mL) was detected in the first sample collected from each pig (Tmax; 5.33 ± 3.27 h, as not all pigs urinated for the first 4 h sample). After 120 h, urine concentrations for all pigs fell below the LOQ of 0.0005 µg/mL. Renal clearance for flunixin was 5.29 ± 2.98 mL/h/kg (Table 2).

**Table 2:** Renal clearance values, % contribution of renal clearance to total systemic clearance (Cl/F) and % total dose excreted in urine following intramuscular administration of 2.2 mg/kg flunixin (*n* = 5) or 0.4 mg/kg meloxicam (*n* = 7)

Renal Clearance					
**Parameter**	**Units**	**Flunixin**		**Meloxicam**	
**Renal Clearance**	mL/h/kg	4.72	(2.98)	0.16	(0.04)
**Renal clearance component of Cl/F**	%	1.31	(0.42)	0.19	(0.06)
**Percent total dose excreted in urine as parent drug**	%	1.39	(0.42)	0.20	(0.06)

#### Liver and Kidney

Flunixin was detected in liver samples from six out of seven pigs at necropsy following two consecutive negative urine samples (96–168 h; concentration range 0.0001–0.0012 µg/g). However, flunixin concentrations in all liver samples were far below the FDA tolerance of 0.03 µg/g [5] and EMA MRL of 0.2 µg/g [14]. Only one kidney sample tested positive for flunixin (right kidney 0.0002 µg/g). Although there is no FDA tolerance level for kidney, this was far below the FSIS confirmatory limit of detection of 0.0125 µg/g [15] and EMA MRL of 0.03 µg/g [14].

### Meloxicam

#### Plasma and ISF

**Fig. 2:**
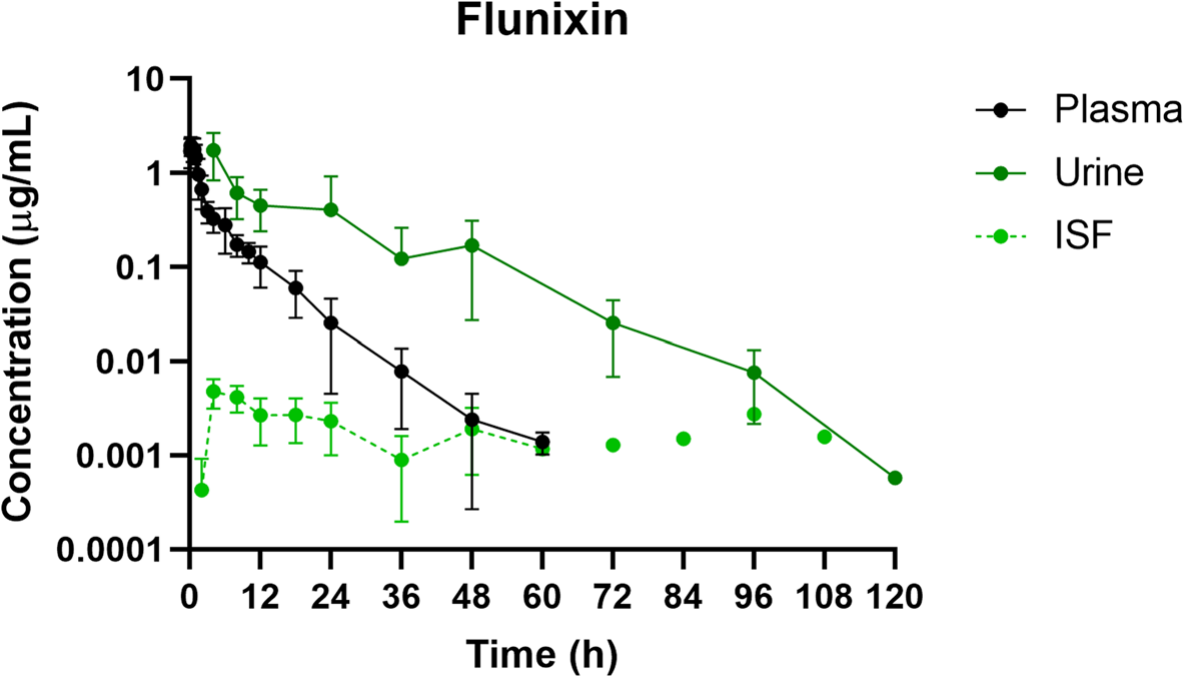
Plasma, urine and interstitial fluid flunixin concentration-time profiles following intramuscular administration of 2.2 mg/kg flunixin to pigs. Data are represented as mean ± standard deviation. Interstitial fluid concentrations shown at 72–108 h are for a single pig only

Mean plasma and ISF meloxicam concentrations over time following a single IM injection of 0.4 mg/kg are presented in Fig. 2. Parameters describing the pharmacokinetics of meloxicam following a single IM injection are presented in Table 1. Meloxicam concentrations in plasma were last detected above the LOQ of 0.001 µg/mL at 36 h. The plasma pharmacokinetics of meloxicam after IM administration were characterized by rapid absorption and a relatively short apparent elimination half-life. In interstitial fluid, the average maximum concentration of meloxicam (Cmax) was 0.0078 µg/mL at 10.5 hours (Tmax) after meloxicam administration.

#### Urine

The highest concentration of meloxicam in urine (Cmax; 0.05 ± 0.01 µg/mL) was detected in the first sample collected from each pig (Tmax; 4.57 ± 1.51 h, as not all pigs urinated for the first 4 h sample). After 48 h, urine concentrations for all pigs fell below the LOQ of 0.001 µg/mL. Renal clearance for meloxicam was 0.17 ± 0.04 mL/h/kg (Table 2).

#### Liver and Kidney

Meloxicam was detected in a single liver sample at necropsy following two consecutive negative urine samples (36–72 h; caudal lobe 0.0054 µg/g, although meloxicam was not detected in samples taken from other lobes of this pig’s liver). While there is no FDA tolerance for meloxicam in pigs, this was below the EMA MRL of 0.065 µg/g [16]. Meloxicam was not detected in any kidney sample.

## Discussion

### Plasma pharmacokinetics

Following intramuscular administration of 2.2 mg/kg flunixin, the peak plasma concentration was reached in 0.22 h, which was slightly less than the previously reported Tmax of 0.61 h and 0.85 h following intramuscular administration to gilts [17] and 6-day-old piglets [18], respectively. The apparent elimination half-life (5.41 h) was similar to previously reported t_1/2_ in 10-day-old piglets following intravenous administration of 2.2 mg/kg and 4.4 mg/kg flunixin (4.82 h and 5.15 h, respectively [19]), but less than previously reported in gilts and 6-day-old piglets administered 2.2 mg/kg intramuscularly (7.49 h and 7.93 h) [17, 18]. Elimination half-life is a hybrid variable and can be altered by flip-flop kinetics and changes in drug distribution and clearance. Variation between studies (including multiple injection sites in the gilt study) could be responsible for these differences in half-lives.

Only one previous study reported the volume of distribution/F (Vd/F) following an intramuscular dose of flunixin, and the reported value was much less than the Vd/F in the present study (0.92 L/kg for 6-day-old piglets [18] compared to 2.88 L/kg in this study). The Vd/F is affected by the bioavailability (F) and the degree of plasma and tissue protein binding, as well as the drug’s lipophilicity [20]. Lipid-soluble drugs such as flunixin have high apparent volumes of distribution, and flunixin has been shown to be highly bound to plasma proteins in pigs (99% [21]). The body fat-to-water ratio increases with age, resulting in increased sequestration of flunixin in adipose tissue [20], which may explain the variation in Vd/F between 6-day-old piglets and the juvenile pigs in the present study, both administered 2.2 mg/kg flunixin intramuscularly.

Following intramuscular administration of 0.4 mg/kg meloxicam, the peak plasma concentration was reached around 0.37 h), which was comparable to previously reported in 8-day-old piglets given a dose of 1 mg/kg (0.50 h [22]), but less than that reported in 5-day-old piglets given 0.4 mg/kg and 2-week-old piglets given 0.6 mg/kg (1.21 h and 1.1 h, respectively [18, 23]. However, the apparent elimination half-life was comparable to most previous reports (3.34 h in this study compared to 2.6 h, 3.94 h and 4.46 h; [18, 22, 23]), except for the elimination half-life reported in sows following an intravenous dose of 0.5 mg/kg (6.15 h; [24]). Despite differences in routes of administration, this difference may suggest that drug elimination may be slower in mature pigs.

Volume of distribution/F in this study for meloxicam (0.41 L/kg) was comparable to that of other studies investigating piglets 5–23 days of age, as well as mature sows, given doses in the range of 0.4-1.0 mg/kg and given via intramuscular or intravenous routes of administration [18, 19, 22, 24, 25].

### Urine pharmacokinetics and renal clearance

As a general rule, NSAIDs are primarily eliminated by hepatic biotransformation, with renal excretion of the parent compound contributing to a small amount of total excretion (< 5% [26]). In this study, the percent of the total dose that was excreted as unchanged parent drug in the urine was low for both flunixin and meloxicam (1.31% and 0.19%, respectively). The total body clearance for each of these NSAIDs was comparable to previous studies [19, 21–23, 25], and represents elimination from the whole body of the parent drug and not its metabolites, including hepatic and renal elimination. The metabolites are predominantly glucuronide or oxidative metabolites of these drugs which were not targeted in our urine analysis, but have been reported in the urine of several species [12, 27]. There is no evidence of phase 2 metabolism of meloxicam in pigs [28], but evidence of several phase one metabolites with 5-hydroxymethyl metabolites being the predominant metabolite. Biotransformation of meloxicam governs meloxicam’s clearance in most species and explains why little of the parent drug is found in the urine and this is not unusual for the oxicams class of drugs. The relative contribution of renal clearance to the overall systemic clearance was low, suggesting that the main route of elimination is hepatic metabolism, although this study is limited in that the metabolites of either NSAID were not measured. Renal clearance of parent flunixin or meloxicam in pigs has not been previously reported, which is a unique feature of this study.

The relationship between plasma and urine flunixin concentrations across all time points indicates that urine flunixin concentrations were higher than those measured in plasma at any given time point, similar to previously reported in both cattle and goats [2, 6], however this was the opposite for meloxicam, with plasma concentrations being similar to or higher than that of the urine, again, similar to previously reported in goats [2].

Our data also allowed us to propose a plausible mechanism of renal clearance of these parent drugs. For example, by assuming glomerular filtration rate (GFR) is 259 mL/h/kg [29] and assuming fraction unbound in plasma, fu = 0.01 for flunixin and for meloxicam, as they are known to be highly bound to plasma proteins, then the clearance by filtration for both drugs can be estimated to be 2.59 mL/h/kg. This value is less than our measured renal clearance of 4.72 mL/h/kg for flunixin, but is greater than our measure renal clearance of 0.16 ml/h/kg for meloxicam. Based on these calculations, one can infer that filtration and active secretion contributed to renal clearance of flunixin but filtration and reabsorption for renal clearance of meloxicam. The latter is consistent with very little meloxicam (< 0.2%) of parent drug appearing in the urine of most species. It should be reiterated here that this pertains to the clearance of the parent dugs and not the metabolites.

### Tissue residues

The FDA established tolerance levels are based on edible tissues, or the slowest depleting organs (target tissues) which often refers to the liver or kidneys. For livestock shows, it is not possible to directly test these target tissues, and plasma samples are not convenient, and urine is tested instead. Detection of drug in urine may be a violation of livestock show rules.

Interstitial fluid (ISF) was collected to create a concentration-time profile in an attempt to reflect the tissue concentrations across multiple time points for each pig. However, these concentrations did not correlate well with plasma or urine concentrations, particularly for flunixin in which the ISF concentrations were prolonged but at a low level. However, the ISF concentrations for both meloxicam and flunixin fell below the LOQ before the urine concentrations.

Flunixin concentrations detected in the liver following negative urine samples were far below the FDA tolerance and present no food safety concerns or violations of livestock show rules. Meloxicam was detected in a single liver sample at necropsy. While there is no FDA tolerance for meloxicam in pigs, this was below the EMA MRL for meloxicam in liver. However, the presence of meloxicam at any level in pig liver in the US would be a violation according to US FSIS.

While the liver is regarded as the main target tissue when examining residues of NSAIDs, this study also measured drug concentrations in the kidneys. Only one kidney sample tested positive for flunixin. Although there is no FDA tolerance level for kidney, the concentration detected was far below the FSIS confirmatory limit of detection. However, limits of detection change as analytical methods improve. Any detectable amount of flunixin in the kidneys would technically be violative. Meloxicam was not detected in any kidney sample, which is consistent with previous work and the renal clearance reported here in the present study.

### Limitations of this study

This study had a number of limitations that should be taken into consideration when reviewing data and applying to livestock show scenarios. As was highlighted earlier, this study did not have a companion intravenous study from which the bioavailability, F, could have been determined, and therefore the clearance and volume of distribution values are approximations and need to be expressed as Cl/F and Vd/F. Nonspecific binding to the ISF probes was not determined. While there is a possibility for drug binding to the ISF probes, these probes are inert materials (polyacrylonitrile) and they were placed 36–48 hours prior to the start of the trial to allow adequate time for equilibration with body fluids. Assessment of drug binding to the probes has been investigated previously for various drugs including NSAIDs such as carprofen and flunixin in other animal species but have not been reported in the literature. In these studies, there was no accumulation when assessed *in vitro*, but we cannot assume this is the same for *in vivo* as there are critical differences in the experimental conditions (for example, the flow rate in vitro is much higher than *in vivo*). This study was focused on estimating the renal clearance of the parent drug and not metabolites, as the primary reason for this study was to determine when parent drug concentrations in urine were below detectable levels so that urine in show pigs are clear of these two drugs. Should livestock show officials target the urinary metabolites for testing, this information may not be applicable. Related to this limitation is the fact that plasma concentration is the driving force controlling all other concentrations including urine concentration, and uncertainty with urine concentrations occurs for many other reasons such as the extent of urine dilution, urinary pH (not reported here), analytical issues, and plasma and urine concentration can be out of phase.

## Conclusions 

The primary purpose of this study was to determine the urinary excretion of two frequently used NSAIDs, flunixin and meloxicam, in pigs and relate these findings to its plasma pharmacokinetics and potential tissue residues. Prior to this study there were data gaps pertaining to the renal clearance of this drug and how long it will take for the parent form of these drugs to clear from urine. While pharmacokinetics parameters from our study were comparable to earlier studies in pigs for these two drugs and a small percentage of the parent drug is cleared in the urine as demonstrated in other species, our study is the first to report the renal clearance of these two NSAIDs in pigs. The data also suggest that the renal clearance mechanism for flunixin was predominantly by active secretion while the predominant clearance mechanism associated with meloxicam was likely by tubular reabsorption. Flunixin urine concentrations were always higher than plasma and ISF concentrations up to 5 days post administration. This was the complete opposite for meloxicam albeit urine levels at 2 days were higher than plasma and ISF levels. When urine levels were negative at 7 days for flunixin and 3 days for meloxicam, the pigs were slaughtered and liver and kidney concentrations were below either the FDA tolerance or the detection level of current US FSIS. However, this study was not able to determine the converse, i.e., whether detectable concentrations of meloxicam or flunixin in the urine correlated with drug concentrations above the tolerance or above detectable levels in the liver. The present study demonstrated that following a single intramuscular dose of 2.2 mg/kg flunixin, the drug should be undetectable in the urine if the label meat withdrawal time (12 days) is followed appropriately. There is no approved label in the US for meloxicam and therefore no established withdrawal time for meloxicam in pigs; however, this study suggests that pigs given a single intramuscular dose 0.4 mg/kg may test positive in urine for up to 2 days post-dose. On the other hand, following negative urine samples, meloxicam may still be detected in the liver (albeit below current FSIS testing limits). As there is no label for meloxicam in pigs, any level detected in the tissues would be considered violative in the US. This study provides useful information that can help livestock show authorities and veterinarians determine an appropriate elimination period for show animals whose urine may be tested prior to competition, and it may help provide data on which to base penalties for detection of flunixin or meloxicam in urine in show pigs.

## Methods

### Animals and housing

North Carolina State University Institutional Animal Care and Use Committee approved this study. All animals were acquired from the North Carolina State University Swine Education Unit and transferred to the North Carolina State University College of Veterinary Medicine, where they were housed individually in metabolism cages (72^o^F), with a 12∶12 light:dark cycle, fed LabDiet 5084 (LabDiet, St. Louis, MO, USA) twice a day and had access to freshwater *ad libitum*. A total of fourteen healthy, castrated, male Yorkshire/Landrace cross pigs (weighing 23.1–35.4 kg) were enrolled to receive either flunixin or meloxicam (n = 7 per treatment group). There is no hypothesis testing involved in this pharmacokinetics study, and therefore a power analysis is not required for estimating sample size as previously described [30]. This study excluded any animals with hernias, diarrhea, lameness or any other clinical signs of disease and inclusion criteria were no prior treatment with flunixin or meloxicam. Pigs appeared healthy on physical examination by lack of any clinical signs. During catheter placement and interstitial fluid probe placement, temperature, heart rate and respiratory rate were monitored, and no abnormalities were noted. Pigs were randomly assigned to metabolism cages at any given trial by individuals not involved in the study. There were three trials consisting of 4 pigs/trial with 2 pigs being treated with flunixin and 2 pigs being treated with meloxicam, and one trial with one pig treated with flunixin and other pig with meloxicam. This allowed us to account for the effect of litter. Investigators were not blinded to sample collection or sample analysis at any stage of the study.

### Catheter and Interstitial Probe Placement

Prior to the start of the study, pigs were moved to individual metabolism cages and allowed 4 days of acclimation. After the adjustment period pigs were sedated using an intramuscular injection of a combination of Telazol® (50 mg/mL tiletamine HCl and 50 mg/mL zolazepam HCl), ketamine (100 mg/mL) and xylazine (100 mg/mL) at a concentration of 0.6 mL/kg body weight. Using sterile technique, an 18 Ga x 15 cm catheter (SA1815; Mila International, Inc., Florence, KY, USA) was inserted into the right jugular vein and sutured to the skin using 2 − 0 monofilament suture and an extension attached.

At the time of catheter placement, an ultrafiltration probe (Canine UF Probe, BASi systems, W. LaFayette, IN, USA) was placed subcutaneously along the epaxial muscles using a previously described technique [31]. The interstitial probe allowed for continuous collection of interstitial fluid (ISF). Pigs were able to recover for 36–48 hours following the placement of instrumentation. During this recovery period, patency of the catheter was maintained by removing the heparin lock (100 mg/mL), flushing the catheter with saline and replacing the heparin lock every 12 hours.

### Drug Administration and Sample Collection

Pigs were administered a single intramuscular dose of either 0.4 mg/kg meloxicam (Meloxicam solution for injection 5 mg/mL, Putney, Inc., Portland, ME, USA), the labeled dose for pigs in Europe, or 2.2 mg/kg flunixin meglumine (Banamine-S®, Merck Animal Health, Summit, NJ, USA), the labeled dose for pigs in the US. The injection location was the neck of the pigs in accordance to the label instructions. Blood samples (3 mL) were collected via the jugular catheter and transferred to lithium heparinized tubes at 0 (baseline), 0.08, 0.17, 0.25, 0.5, 0.75, 1, 1.5, 2, 3, 4, 6, 8, 10, 12, 18, 24, 36, 48, 60, 72, 84, 96, 108, 132, 156 and 180 h post-administration of flunixin or meloxicam. Blood samples were centrifuged at 3500 x g and the plasma collected for analysis of total drug concentrations.

Interstitial fluid samples were collected via the preplaced collection probes at 0 (baseline), 2, 4, 6, 8, 10, 12, 18, 24, 36, 48, 60, 72, 84, 96, 108, 132, 156 and 180 h post-dose and weighed to determine the volume collected. At the end of the experiment, the ISF probe was removed and the tubing length measured. A lag time for the ISF collection was calculated to account for the time taken for the sample to travel along the ISF probe tubing. Interstitial fluid was used to quantify the free (protein unbound/pharmacologically active portion) drug concentrations in the tissues.

In order to determine drug concentrations in urine, animals were housed individually in metabolism cages to allow for collection of urine samples. Urine samples were collected at 0 (pretreatment), 4, 8, 12, 24, 48, 72, 96, 120, 144 and 168 h after drug administration. Urine was collected via a tray under the metabolism cages with a spout at the front of the cage and a stainless-steel bucket underneath to catch the urine and limit fecal contamination. Further details of the sample collection and calculation are described in our previous study in goats by Bublitz et al. [2]. All plasma, ISF and urine samples were frozen at -80 ºC prior to analysis.

### Tissue collection

After two consecutive negative (drug-free) urine samples (36–72 h for meloxicam, and 96–168 h for flunixin), each pig was euthanized. In order to minimize stress associated with euthanasia, the pigs were first sedated via intramuscular injection of 50:50 ketamine (100 mg/mL) and xylazine (100 mg/mL), equivalent to a final dose of 2.2 mg/kg ketamine and 2.2 mg/kg xylazine. After sedation, Euthasol® was administered through the jugular vein catheter at a dose equivalent to 85.9 mg/kg pentobarbital sodium and 11 mg/kg phenytoin sodium. Several biopsy punches were taken from each lobe of the liver and the entire left and right kidneys were taken from each pig in order to analyze tissues when the drug was no longer detectable in urine. These tissue samples were frozen at -20^o^C until analysis.

### Drug Analysis

#### Plasma and Urine Sample Preparation

Flunixin and meloxicam plasma and urine samples were prepared using solid-phase extraction prior to UPLC-MS/MS analysis. Samples (300 µL) were pretreated with 300 µL of 4% phosphoric acid and vortexed for 10 seconds. Then, 500 µL of this pretreated sample was loaded onto an Oasis 1 mL 30 mg PRiME HLB cartridge (Waters Corp.), washed with 1 mL of methanol, and eluted from the cartridge with 500 µL of 90:10 (vol/vol) acetonitrile:methanol. The eluate was filtered through a 0.2 µm PTFE Whatman Mini-UniPrep Syringeless Filter vial (GE Healthcare UK Limited., Buckinghamshire, UK) and then injected onto the UPLC-MS/MS system.

#### Tissue Sample Preparation

For all kidney and liver samples, a 0.1 g sub-sample was weighed into a 2-mL bead mill tube containing 2.8‐mm ceramic beads (Fisher Scientific, Hampton, NH, USA). Then, 1 mL of acetonitrile was added to the tube and the contents homogenized 3 times for 15 seconds during each cycle at a speed of 5 m/s for kidney, and 4 m/s for liver, with a 10 second rest between cycles (BeadMill24, Fisher Scientific). Following homogenization, the tubes were centrifuged at 10,000 x g for 5 minutes. Then, 800 µL of supernatant was transferred to a 16 × 100 mm borosilicate glass tube containing 800 µL of acetonitrile and 400 µL of water. This mixture was vortexed gently for 10 seconds and then eluted through a 3 mL Captiva EMR-Lipid cartridge (Agilent Technologies, Inc., Santa Clara, CA, USA). The eluate was then evaporated to dryness at 55 °C for 25 minutes. The sample was reconstituted in 300 µL of 1:1 acetonitrile:water, vortexed for 30 seconds, and the contents transferred a 0.2 µm PTFE Whatman Mini-UniPrep Syringeless Filter vial and then injected onto the UPLC-MS/MS system.

#### UPLC-MS/MS Conditions

All samples were quantified by ultra-high-pressure liquid chromatography (UPLC) with mass spectrometric (MS/MS) detection (Waters Corp., Milford, MA, USA). The UPLC-MS/MS system consisted of a Xevo TQD tandem quadrupole mass spectrometer (Waters Corp.)

For flunixin samples, separation was achieved with a 2.1 mm x 100 mm, 1.7 µm Waters Acquity BEH Phenyl column (Waters Corp.). A gradient was used, and the initial mobile phase was 0.1% formic acid in water: 0.1% formic acid in acetonitrile (70:30 v/v) with a flow rate of 0.4 mL/min for the first 2.5 minutes. The mobile phase then switched to (10:90 v/v) from 2.5 min − 3.5 min. For the last 1.5 min of the run, the mobile phase was (70:30 v/v). The MS/MS was run in ESI + mode. The quantification trace used was 297 → 279. Column temperature was 35 °C and sample temperature was ambient.

For meloxicam samples, separation was achieved with a 2.1 mm x 50 mm, 1.7 um Waters Acquity BEH C18 column (Waters Corp.) A gradient was used, and the initial mobile phase was 0.1% formic acid in water: 0.1% formic acid in acetonitrile (65:35 v/v) with a flow rate of 0.4 mL/min for the first minute. The mobile phase then switched to (10:90 v/v) from 1.0 min to 1.1 min. For the last 1.9 min of the run, the mobile phase was (65:35 v/v). The MS/MS was run in ESI + mode. The quantification trace used was 352.043 → 115. Column temperature was 35 °C and sample temperature was 10 °C.

Validation standards were prepared over a linear range for each matrix (plasma, urine, kidney and liver) and were used to construct calibration curves. For the inter-day accuracy and precision, standards were repeated over 3 days. The concentrations analyzed varied by drug and by matrix and are shown in Table 3 below.

**Table 3:** Concentrations and replicates used for the assay validation

**Drug**	**Matrix**	**# of concentrations**	**Concentrations (μg/mL)**
Flunixin	Plasma	6	0.0005, 0.001, 0.01, 0.1, 0.5, 1
	Urine	7	0.0005, 0.001, 0.005, 0.01, 0.05, 0.1, 0.5
	ISF	6	0.0005, 0.001, 0.002, 0.005, 0.01, 0.05
	Liver	6	0.0001, 0.0005, 0.001, 0.01, 0.05, 0.1
	Kidney	5	0.0005, 0.001, 0.01, 0.05, 0.1
Meloxicam	Plasma	7	0.001, 0.005, 0.01, 0.05, 0.1, 0.5, 1
	Urine	6	0.005, 0.01, 0.05, 0.1, 0.5, 1
	ISF	6	0.0005, 0.002, 0.005, 0.01, 0.025, 0.05
	Liver	5	0.005, 0.01, 0.1, 0.5, 1
	Kidney	6	0.005, 0.01, 0.05, 0.1, 0.5, 1

All calibration curves were linear with a R^2^ value of 0.99 or higher. Limit of quantification, inter-day accuracy and inter-day precision are presented in Table 4 for each analytical method.

**Table 4:** Limit of quantification (LOQ; g/mL for fluids or g/g for tissues), inter-day accuracy (%) and inter-day precision (%) for analytical methods

Sample Analysis Parameters						
**Drug**	**Tissue**	**LOQ**	**Accuracy (%)**		**Precision (%)**	
		**µg/mL or µg/g**	**Mean**	**±SD**	**Mean**	**±SD**
Flunixin	Plasma	0.0005	107	(6)	5	(3)
	ISF	0.0005	99	(7)	6	(5)
	Urine	0.0005	103	(5)	6	(3)
	Liver	0.0001	100	(5)	5	(2)
	Kidney	0.0001	100	(7)	8	(5)
Meloxicam	Plasma	0.001	100	(5)	6	(5)
	ISF	0.001	97	(6)	6	(2)
	Urine	0.001	104	(5)	4	(2)
	Liver	0.005	100	(5)	7	(2)
	Kidney	0.005	100	(4)	8	(2)

*SD *Standard deviation

### 4.6 Pharmacokinetic analysis

A noncompartmental analysis of drug plasma concentration vs. time profiles was performed with Phoenix WinNonLin software (version 8.0; Certara, Princeton, NJ, USA). The area under the plasma concentration–time curve from time zero to infinity (AUC0→∞; h*µg/mL) was calculated by linear log trapezoid method. The AUC0→∞ was used to calculate clearance per fraction absorbed (Cl/F; L/h/kg) and half-life (T1/2; h). The volume of distribution (per fraction absorbed) (Vd/F; L/kg) was also calculated. Peak concentration (Cmax; µg/mL) and time at which maximum concentration occurs (Tmax; h) in plasma and urine was taken directly from the data from each pig.

Individual renal clearance values were estimated for each pig using the following equation: Renal Clearance (mL/h) = [(Ae/AUC)] [32], where Ae is the cumulative amount of drug excreted unchanged in the urine and AUC is the area under the plasma concentration-time curve to infinity. These values were then corrected for body weight for comparison with the total body clearance and reported as mL/h/kg in the Table 2.

## Abbreviations

EMA: European Medicines Agency
FDA: Food and Drug Administration
FSIS: Food Safety and Inspection Service
ISF: Interstitial fluid
LOQ: Limit of Quantification
MRL: Maximum residue limit
NSAID: Nonsteroidal anti-inflammatory drug
SD: Standard deviation
UPLC-MS/MS: Ultra performance liquid chromatography-tandem mass spectrometer
USDA: United States Department of Agriculture

## Availability of data and materials

The datasets used and/or analyzed during the current study are available from the corresponding author on reasonable request.
